# Tribological Behavior of Ionic Liquid with Nanoparticles

**DOI:** 10.3390/ma14216318

**Published:** 2021-10-22

**Authors:** Thi-Na Ta, Shin-Yuh Chern, Jeng-Haur Horng

**Affiliations:** Department of Power Mechanical Engineering, National Formosa University, Yunlin 63201, Taiwan; natt.mta@gmail.com (T.-N.T.); kevindga@nfu.edu.tw (S.-Y.C.)

**Keywords:** ionic liquid, zinc oxide, copper oxide, anti-wear, anti-friction

## Abstract

This research aims to formulate a new lubricant containing oxide nanoparticles for enhancing anti-wear ability and reducing friction. Different concentrations of copper oxide (CuO) and zinc oxide (ZnO) nanoparticles were separately added to an ionic liquid, methyltrioctylammonium bis(trifluoromethylsulfonyl)imide [N1888] [NTf2], to formulate the tested lubricants. The tribological properties of the lubricants were tested by performing ball-on-disc wear tests on a tribotester (MTM, PCS Instruments). The results show that both the CuO and ZnO nanoparticles can increase the friction reduction ability of the ionic liquid when used as a neat lubricant. The anti-wear characteristic of the ionic liquid is increased by adding ZnO nanoparticles but decreased by adding CuO nanoparticles. The best tribological performance observed for the concentration of 0.2 wt% ZnO, with the wear scar diameter is reduced by 32% compared to the pure ionic liquid. The results of SEM/EDX analysis on the worm morphologies show different lubrication mechanisms of the nanoparticles in the [N1888] [NTf2], which are tribo-sintering for CuO nanoparticles, and third body with pure rolling effect for ZnO nanoparticles.

## 1. Introduction

Ionic liquids (ILs) are salts composed of organic cation and organic or inorganic anions that commonly melt below 100 °C. ILs exhibit numerous features including environmentally friendly materials, high thermo-oxidative stability, non-flammability, good chemical stability, and high viscosity. ILs were first used as lubricants by Ye et al. [1]. Since this report, numerous studies on ILs have been conducted for many kinds of friction materials and lubricants. ILs can react with metal surfaces forming a tribofilm, depending on the materials of contact surfaces and the elements of anions and cations in ILs. Typical anions in ILs include tetrafluoroborate (BF4), hexafluorophosphate (PF6), and bis-trifluoromethanesulfonimide (NTf_2_). The main types of cations are imidazolium, phosphonium, ammonium, and pyrrolidinium. ILs with an imidazolium cation increase the alkyl chain length, which can prevent direct metal-to-metal contact [2]. Comparing to imidazolium-based ILs, ILs with phosphonium, ammonium, and pyrrolidinium cations present lower friction and better anti-wear characteristics. Previous studies pointed out that imidazolium-based ILs with [PF6] and [BF4] anions exhibit good tribological behavior, but the chemical components of those anions contain a rich fluorine compound that can be highly corrosive and toxic. Thus, recent studies on ILs emphasize [NTf2] anion-based ILs according to their excellent tribological performance. Methyltrioctylammonium bis(trifluoromethylsulfonyl)imide [N1888] [NTf2] is considered a biocompatible IL and less toxic compared to the other halogen anion-based ILs [3]. To complement the studies on [NTf2] anion-based ILs, this study investigates the tribological behavior of the [N1888] [NTf2] when blended with oxide nanoparticles and used as neat lubricant in steel–steel contact pairs.

In most applications of ILs, they can be used as additives [4,5] or neat lubricants [6]. Kreivaitis et al. [7] showed that the addition of ILs in water could significantly reduce friction and wear. Since ILs exhibit outstanding performance in terms of friction reduction, anti-corrosion, and wear resistance ability, they become novel lubricant additives in many industrial applications, such as micro-electromechanical machines (MEMs) [8], engine lubrication [9], and metal cutting fluid [10]. Up to now, there have been many review articles summarizing the applications of ILs for different research areas [11,12]. However, how to improve the tribological properties of ILs has not been carried out in recent studies. Hence, the [N1888] [NTf2] was formulated with nanoparticles to enhance its tribological performance in the present study.

Recently, many published research articles have reported the applications of nanoparticles in the field of tribology. Blending the nanoparticles into lubricants can significantly enhance the anti-friction and anti-wear behaviors of lubricating oils. Among all nanoparticles, copper oxide (CuO) and zinc oxide (ZnO) have been widely researched due to their excellent tribological properties. The addition of CuO nanoparticles could reduce friction and wear when they were blended into different types of lubricating oils [13,14,15,16,17,18,19,20,21]. For instance, Kumar et al. [14] presented that the CuO nanoparticles could be mixed with the zinc dialkyldithiophosphates (ZDDP) additive to increase the wear resistance ability of vegetable oil. The CuO nanoparticles were also used as additives to improve the anti-wear and extreme pressure performances of coconut oil [15], palm oil [16], and canola oil [17]. Moreover, the CuO nanoparticles were compatible with engine oils and resulted in significant friction reduction [18,19]. A reduction in friction coefficient by 37.9%, 42.9%, and 14.6% for three different concentrations of 0.2, 0.5, and 1.0 wt%, respectively, was observed when CuO nanoparticles were added to a mineral-based lubricant [20]. Reference [21] showed that the addition of CuO nanoparticles to grease could decrease the friction coefficient and wear scar diameter significantly. 

Similar to CuO, ZnO nanoparticles have been used as additives for improving the tribological behavior in many types of lubricating oils [22,23,24,25,26]. Mana et al. [22] reported that the addition of ZnO nanoparticles in mineral-based oil showed better friction reduction ability than the commonly used anti-wear additives ZDDP. The ZnO nanoparticles combined with diesel oil could improve the thermophysical properties of diesel oil [23,24]. Mello et al. [25] made a comparison of the tribological performance of ZDDP, CuO, and ZnO when used as additives in mineral and synthetic oils. The results showed that the additions of CuO and ZnO nanoparticles could reduce friction more effectively than ZDDP additives. 

Generally, zinc oxide nanoparticles are considered as “green materials” with excellent thermal stability, as well as antibacterial and anti-corrosion properties, so they are used in many industries such as textiles [27], medical fields [28], and tribology [29,30]. By taking the advantages of ZnO and CuO nanoparticles, such as environmental friendliness, reducing friction, and anti-wear properties, this study selects CuO and ZnO nanoparticles to be used as additives in the IL [N1888] [NTf2].

The improvement in tribological properties of lubricants when the IL, CuO, and ZnO nanoparticles are separately used as additives has been studied in previous research. However, the tribological behavior of the IL containing these oxide nanoparticles, when used as neat lubricants in a steel–steel contact, is presented for the first time in this paper. A comparison of friction coefficients, wear widths, formation of the tribofilms’ thickness, and chemical components on wear surfaces were made to evaluate the effects of oxide nanoparticles on the tribological behavior of the IL. In addition, this study discusses the anti-wear mechanism by the chemical interaction between friction pair materials, nanoparticles, and ionic liquid. 

## 2. Experimental Description

**Figure 1:**
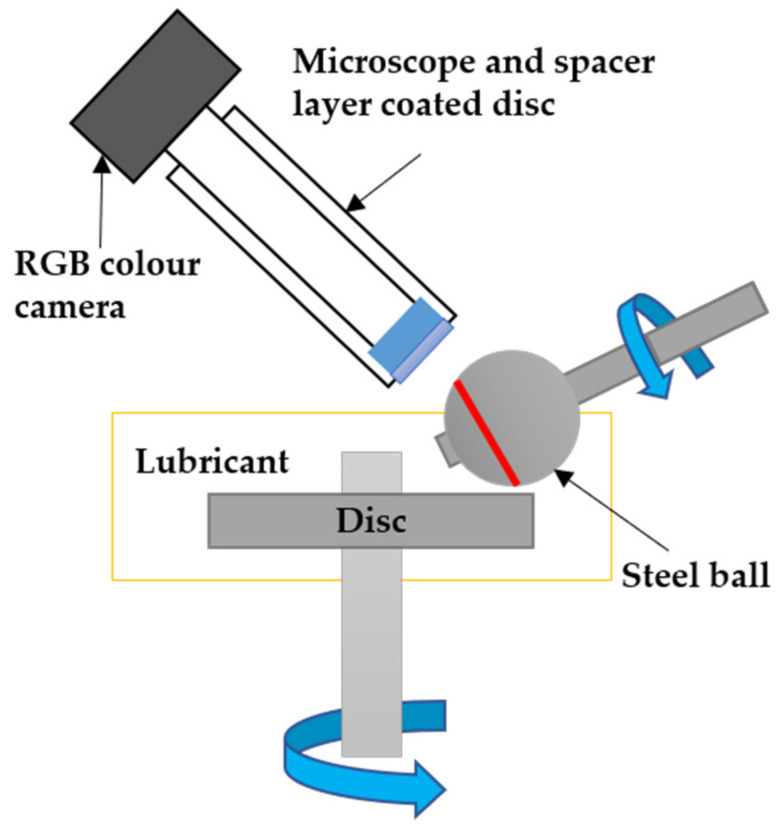
Schematic representation of MTM-SLIM test.

A mini traction machine (MTM) tribotester with a spacer layer imaging (SLIM) technique was used to observe the influences of adding different concentrations of oxide nanoparticles, CuO and ZnO, in the IL [N1888] [NTf2] on friction, wear, and tribofilms; thickness of a steel–steel contact. The MTM generates a sliding/rolling contact between a steel ball and steel disc, as shown in Figure 1. Film thickness formed on the ball’s surface was measured in situ within the test rig using the SLIM attachment. By one MTM-SLIM step, also called mapper step, a color optical interference image of the ball surface is automatically acquired. Through SLIM offline analysis program, the thickness of film formed on the ball is analyzed from the SLIM images. In order to take a color image in the SLIM step, the ball is unloaded from the disc and loaded against a glass window under a stationary position. This procedure is controlled automatically by the MTM-PC software, so the test specimens are maintained in test chamber during experiments. 

The material properties of the specimens used for wear tests on the MTM machine are shown in Table 1. Both the ball and disc used were AISI 52100 steel materials with a roughness Ra of 0.02 μm. The hardness of the disc was made lower than the ball’s hardness to ensure that more wear and chemical reaction occurred on the disc surface during the wear process. The chemical composition properties of the specimen materials are shown in Table 2.

The IL [N1888] [NTf2], commercially available from Sigma–Aldrich, Darmstadt, Germany [32], was used as a pure lubricant. Two oxide nanoparticles, CuO and ZnO, purchased from Gredmann, were blended into the IL at two different concentrations of 0.2 and 0.5 wt%. The size of both oxide nanoparticles was 30 nm. The main properties of the IL are presented in Table 3. The nanoparticles were dispersed into the ionic liquid by using a magnetic stirrer for 2 h. The mixed lubricants including the IL and the oxide nanoparticles were used as neat lubricants in the wear tests. A small amount of the tested lubricants (200 μL) was used to wet the disc surface. Before the wear process, the temperature of the test chamber was preheated to 60 °C, then controlled to keep it stable at this temperature during the wear process. The wear tests were performed for a 120 min duration at a constant sliding speed of 100 mm/s. A load of 40 N, corresponding to maximum Hertzian pressure 1.0 GPa, was applied for the contact between the ball and disc. MTM-SLIM steps were conducted at the initial step, 60 min, and 120 min of the wear process to acquire the growth rate of the tribofilm thickness on the ball surface. At least three experiments were performed under the same test conditions of speed, load, sliding–rolling ratio, and test chamber temperature. After the wear tests, the worm morphologies were analyzed using optical microscopy (OLYMPUS STM6) and scanning electron microscopy (SEM, JSM-7610FPlus). Energy dispersive X-ray spectroscopy (EDX) was performed to study the chemical composition of wear scars on the disc surface.

## 3. Results and Discussion

### 3.1. Friction and Wear Behaviors

#### 3.1.1. Friction Coefficients

**Figure 2:**
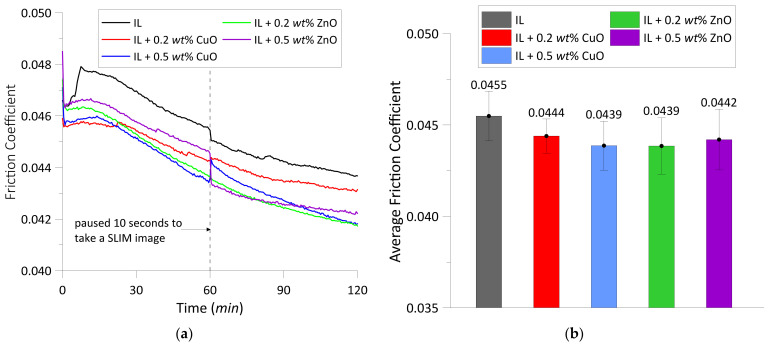
(**a**) The variation of friction coefficient vs. test time; (**b**) average friction coefficient calculated in the 2 h test.

The friction coefficient data presented in Figure 2a were recorded in situ during the wear tests. The wear process was paused 10 s to take a SLIM image at the elapsed time of 60 min. This caused the change of friction coefficients because the ball was loaded to contact with the camera window, and then re-contacted with the disc at the applied load of 40 N. Many research articles have shown that extremely low friction can be observed in ILs’ lubricated contact pairs. In this study, low friction coefficients were also observed for all tested lubricants with/without the nanoparticles. The friction coefficients increased during the first 10 min, then decreased for the remaining time of the wear process. During the first 10 min of rubbing time, the running-in period, the peaks of friction coefficient in the tests with the CuO and ZnO were lower than that of the test with pure ionic liquid. It is speculated that the appearance of the nanoparticles could reduce direct contact between the ball and disc and compensate for nanodefects on the original contact surfaces. This observation is similar to the reduction in the initial friction coefficient during the running-in period, as the CuO and ZnO nanoparticles were added to lubricating oils [33].

From the averaged friction coefficient values shown in Figure 2b, IL + 0.5 wt% CuO and IL + 0.2 wt% ZnO exhibit the lowest friction coefficient value of 0.0439. Compared to the test lubricated with pure IL, the friction coefficient reduced by approximately 3.5% when 0.5 wt% CuO and 0.2 wt% ZnO nanoparticles were blended to the IL. For the tests lubricated with IL + 0.2 wt% CuO and IL + 0.5 wt% ZnO, the friction coefficients were reduced by approximately 2.4% and 2.9%, respectively. The decrease in friction coefficients as adding CuO and ZnO nanoparticles to the IL can be explained by two kinds of lubrication mechanisms, named tribo-sintering and third body mechanisms [19,25,34]. The oxide nanoparticles may deposit or be tribo-sintered on wear surfaces to reduce metal-to-metal contact and act as load-bearing areas.

**Figure 3:**
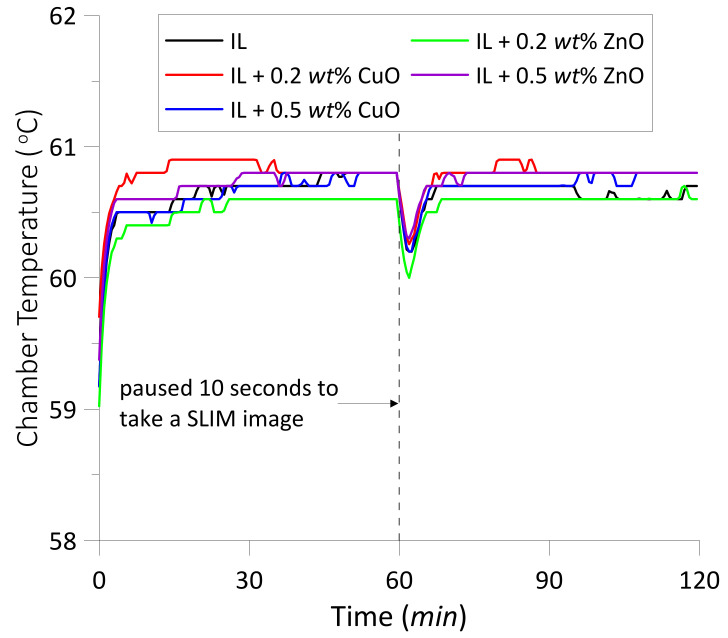
The variation of chamber temperatures during the wear tests.

The viscosity behavior of ILs is strongly dependent on temperature and pressure [35]. The change in the IL viscosity can affect the friction as well as the nanoparticles dispersion in the IL. As mentioned above, the specimens and lubricants remained in the test chamber during the wear process, and the temperature of chamber was controlled at 60 °C. Thus, the change in chamber temperatures was tiny for all tests with/without the nanoparticles as shown in Figure 3. This has very little effect on the lubricant temperature, so the change in the IL viscosity to temperature is negligible. Furthermore, the sliding velocity and load were constant throughout the experiments. Therefore, the variation of friction coefficient in Figure 2 is considered to be independent of the working conditions and temperature.

#### 3.1.2. Wear

**Figure 4:**
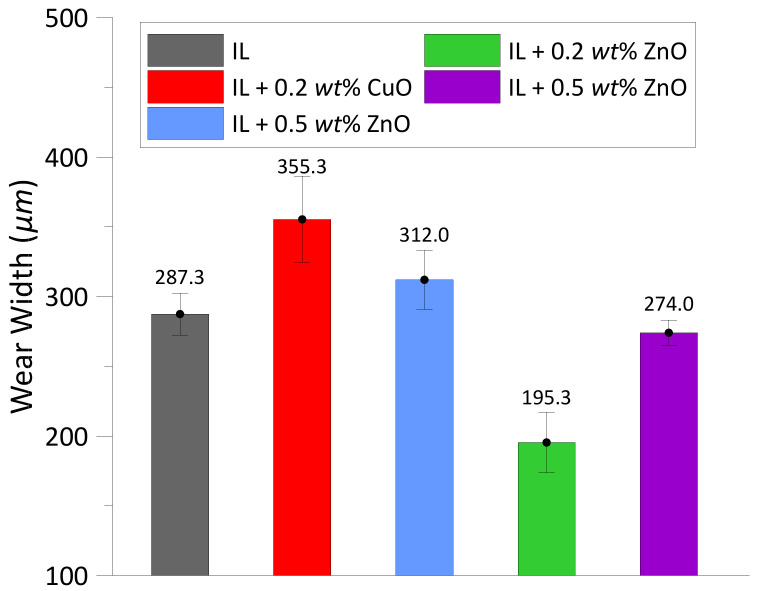
Average widths of wear scars on the disc.

The average wear widths of the disc for all test lubricants are shown in Figure 4. The CuO and ZnO nanoparticles showed different properties when they were blended into the ionic liquid. The addition of ZnO nanoparticles to IL could reduce the wear scar width better than the pure IL. In the test lubricated with 0.2 wt% ZnO, the width of wear scar was reduced by appropriately 32%. Nevertheless, the CuO particles did not exhibit good results in wear width reduction, as they increased the wear width to approximately 8.6% and 23.7% corresponding to the nanoparticle concentrations of 0.5 and 0.2 wt%, respectively. From the experimental results shown in Figure 2b and Figure 4, it can be seen that the best tribological behavior was observed at the test of IL + 0.2 wt% ZnO.

**Figure 5:**
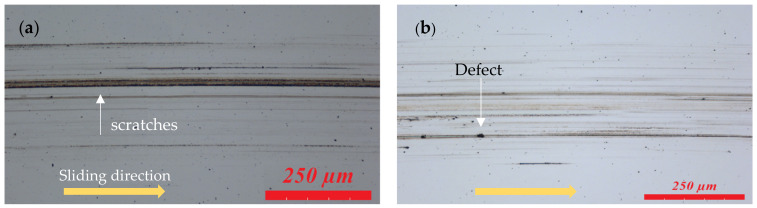

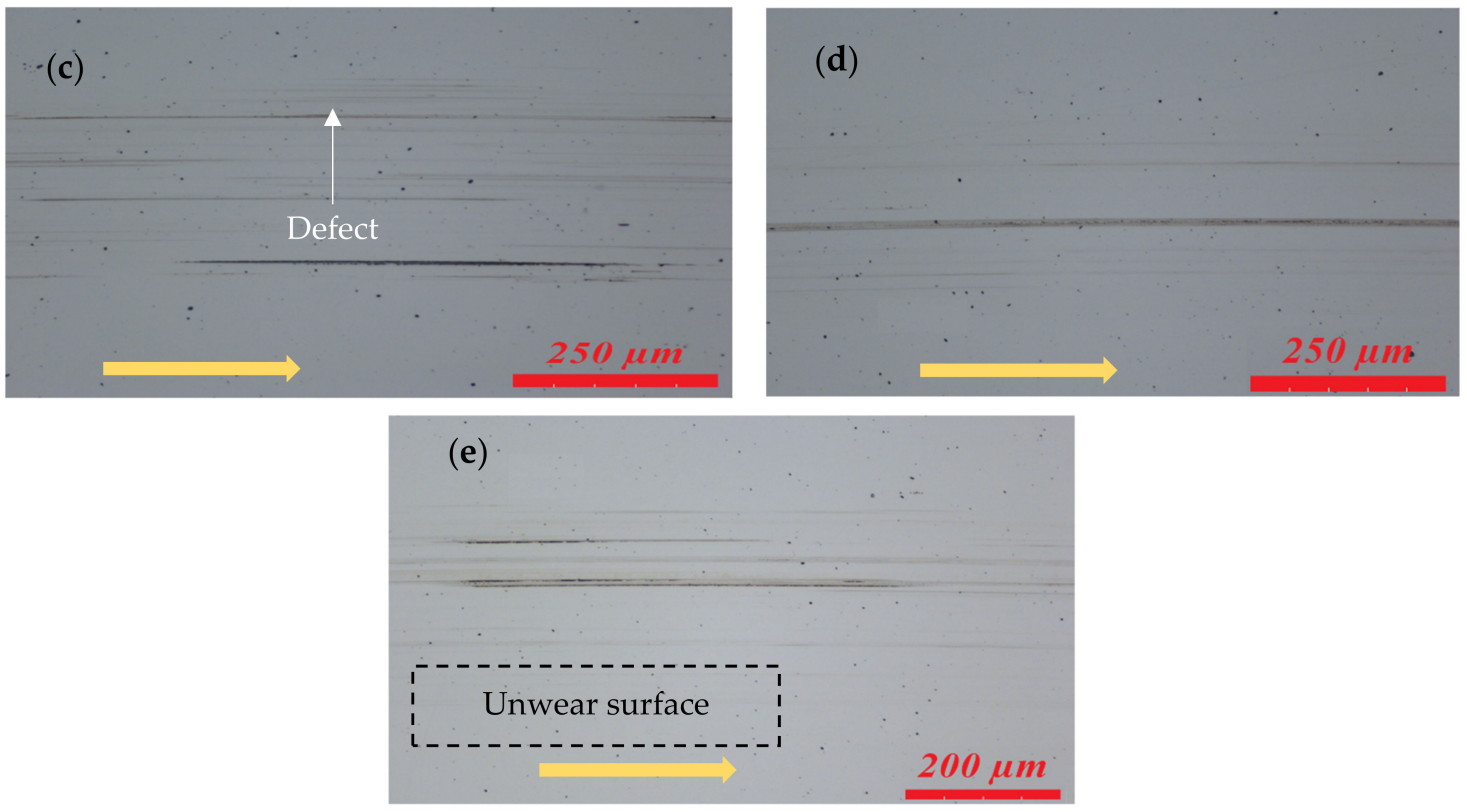
Optical micrographs of the wear surfaces on the discs from tests lubricated with the ionic liquid and different concentrations of CuO and ZnO nanoparticles: (**a**) IL, (**b**) IL + 0.2 wt% CuO, (**c**) IL + 0.5 wt% CuO, (**d**) IL + 0.2 wt% ZnO, and (**e**) IL + 0.5 wt% ZnO. The yellow arrows on the images show sliding directions in the wear tests.

The morphology of the wear scars on the disc surfaces for the tested lubricants are shown in Figure 5; more scratches and defects on worm surfaces were observed for the test lubricated with pure IL. For the tests of CuO and ZnO nanoparticles, light scratches on wear scars are demonstrated for the low friction coefficient in Figure 2. Because the shape of CuO and ZnO nanoparticles is near spherical, they can produce rolling effects between contact surfaces (ball-to-disc) for reducing friction and preventing damage on the surfaces.

### 3.2. Tribofilm Thickness

**Figure 6:**
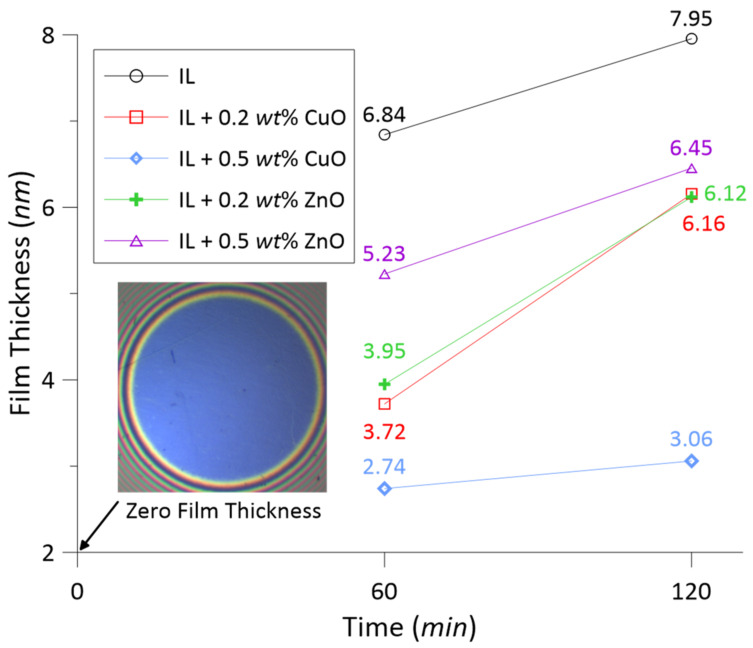
Measured film thickness at wear track on the ball.

Figure 6 shows the tribofilm thickness measured on the ball-rubbed tracks obtained from SLIM images. Film thickness increased as test time duration increased for all tested lubricants with/without the oxide nanoparticles. During the wear process, chemical reactions between the disc surface and lubricants may occur. Consequently, protective tribofilms may be found on the wear surfaces. However, the oxidation process, the appearance of wear debris and nanoparticle additives influenced the formation and sustainability of the tribofilms. Moreover, the concentrations and types of oxide nanoparticles are important factors affecting the formation and growth of protective films. At the concentration of 0.2 wt%, both CuO and ZnO nanoparticles demonstrated similar results of film thickness. As increasing the concentration of nanoparticles to 0.5 wt%, the film thickness increased in the test with ZnO, while it decreased in the test with CuO.

Comparing to the test of pure IL lubricant, the addition of CuO and ZnO nanoparticles caused a decrease in thickness of films as indicated in Figure 6. The decrease in film thickness can be explained due to the electropositive characteristic of the metal nanopaticles and friction pair materials; they hardly react with each other to form a protective layer. For the tests of CuO and ZnO nanolubricants, it was observed that the measured film thickness represented the same trend as the wear width (see Figure 4). In other words, when the film thickness increased, the wear width increased. The thicknesses of tribofilms were very low, only a few nanometers, so these tribofilms did not play a key role in reducing friction and wear. Hence, the anti-wear mechanism of the ionic lubricant with CuO and ZnO nanoparticles is not evaluated by the formation of protective films on wear surfaces.

**Figure 7:**
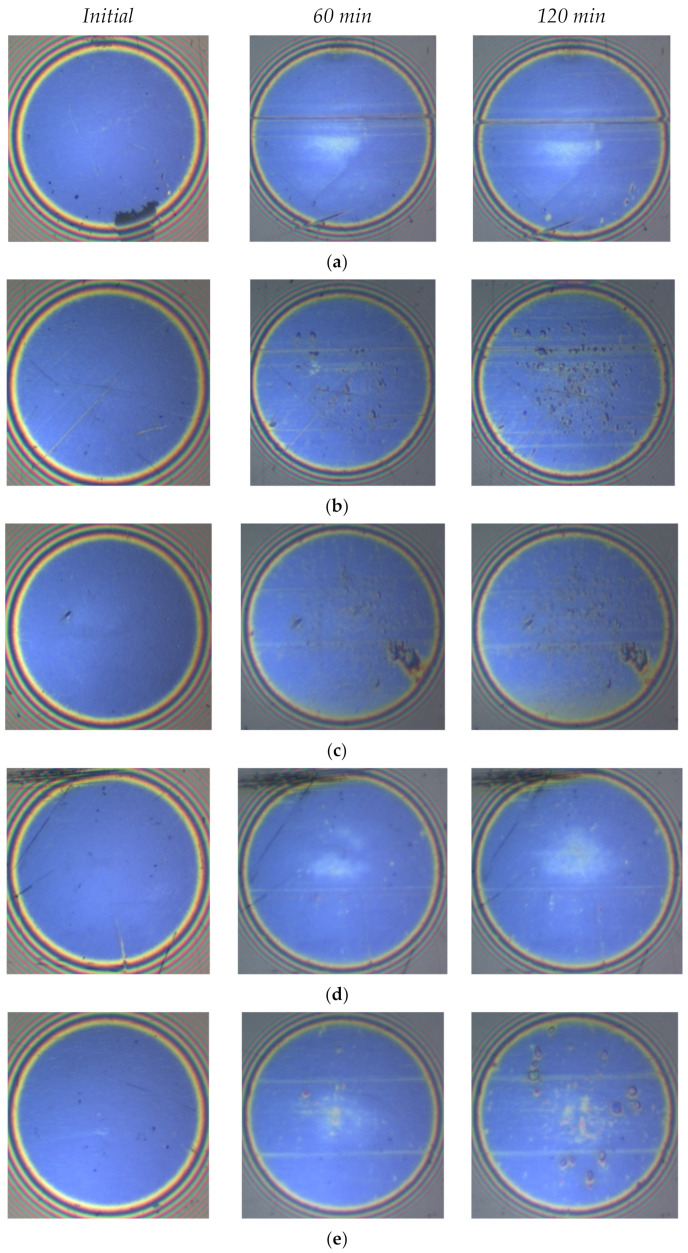
Series of interference images for different concentrations of nanoparticles after increasing rubbing time: (**a**) IL, (**b**) IL + 0.2 wt% CuO, (**c**) IL + 0.5 wt% CuO, (**d**) IL + 0.2 wt% ZnO, and (**e**) IL + 0.5 wt% ZnO.

The interferometric images of the ball-rubbed tracks from tests lubricated with the IL and different concentrations of CuO and ZnO nanoparticles are presented in Figure 7. The first SLIM image of each test was taken before the wear process when the ball surface was completely clean and not lubricated with tested lubricants. The evolution of film thickness shown in Figure 6 was obtained from these images. More severe scratches on the ball surface were seen in the test of ionic liquid, which showed the same surface morphology as Figure 5a. The SLIM images with both 0.2 wt% and 0.5 wt% CuO nanoparticles showed dark regions on the ball surface. It is speculated that these dark regions were the results of chemical reactions between CuO nanoparticles, [N1888] [NTf2], and the metal surface to form tribofilms, or the CuO nanoparticles were deposited on the surface. Nevertheless, the measured film thickness decreased with increasing the concentration of CuO. Therefore, the dark regions represent the deposition of CuO nanoparticles on the contact surfaces. This deposition can compensate for the mass loss on the contact surfaces by the “mending effect”. This mechanism of CuO nanoparticles was reported in previous research, where CuO nanoparticles were used as an additive in synthesis oil [25].

In the tests lubricated with ZnO, the color at the center of interferometric images was found to be quite similar to that of the test with pure IL. It suggests that ZnO combined with the IL did not significantly affect the tribofilms’ properties formed by the IL. However, as the concentration of zinc oxide nanoparticles increased, colored areas appeared on the wear surface of the ball that were different from the color of tribofilms formed by the IL. The increase in the tribofilms’ thickness in the test of IL + 0.5 wt% ZnO was shown by the darker colors in the interferometric images.

### 3.3. Surface Analysis

**Figure 8:**
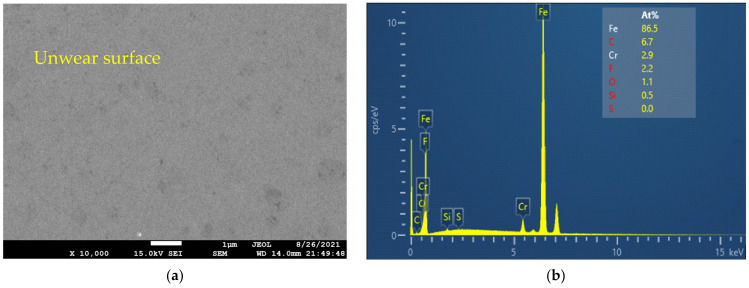
(**a**) SEM image and (**b**) EDX analysis of the unwear surface on the disc.

Chemical components on disc surfaces after the wear test were analyzed by SEM/EDX at three locations, including unwear surfaces, scratches, and defects. Before any test of surface analysis, the specimens were cleaned with ethanol in an ultrasonic bath. Figure 8 shows an SEM image and EDX analysis results of the unwear surface. Although the unwear surface was not abraded by the contact between the ball and disc, the appearance of the fluorine component from the [N1888] [NTf2] showed that chemical reactions may occur between the [NTf2] anion and the metal surfaces upon heating. The chemical element fluorine may affect the corrosion process on the metal surfaces depending on its concentration [36].

**Figure 9:**
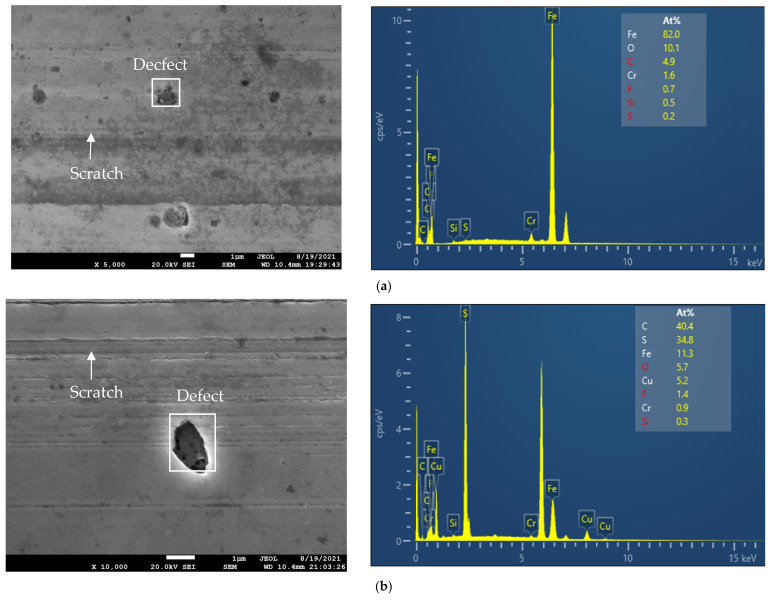

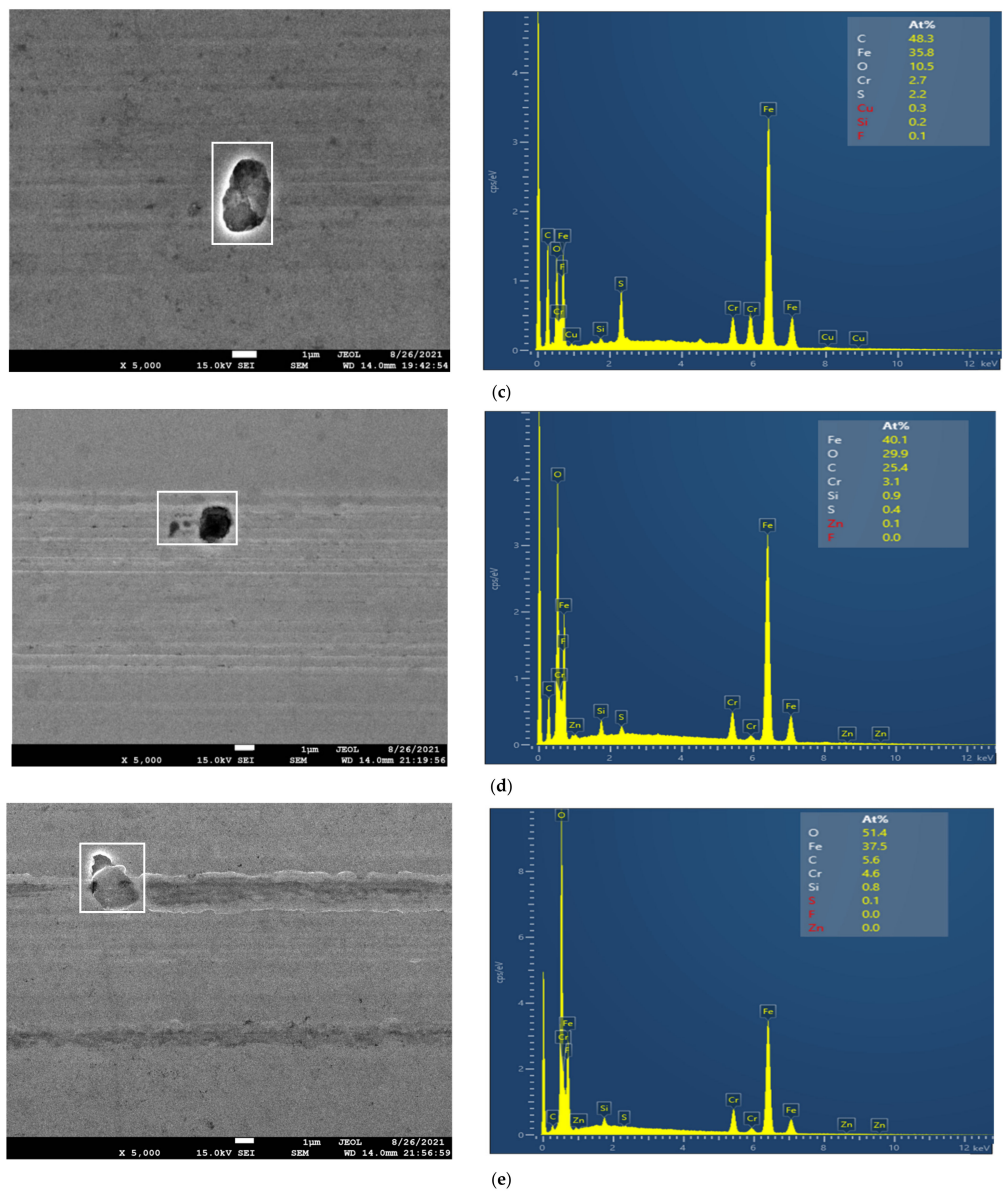
SEM micrographs of worn surface (left) and EDX analysis of defects (right) for the tests lubricated with the IL and different concentrations of nanoparticles: (**a**) IL, (**b**) IL + 0.2 wt% CuO, (**c**) IL + 0.5 wt% CuO, (**d**) IL + 0.2 wt% ZnO, and (**e**) IL + 0.5 wt% ZnO.

SEM/EDX surface analysis of the rubbed surfaces on discs from the tests lubricated with the IL and different concentrations of CuO and ZnO nano-oxides are shown in Figure 9. The chemical composition of worm surfaces on the disc was analyzed at two different points, defects, and scratches. The survey spectra and the weight percentages of chemical compositions on the defects are represented by the figures on the right side of the corresponding SEM images. The distribution of chemical components on the scratch line for all tested lubricants is summarized in Table 4.

Both of the oxide nanoparticles exhibited different lubrication mechanisms when they were added to the [N1888] [NTf2]. The presence of copper in the EDX results, shown in Figure 9b,c and Table 4, indicates that the CuO nano-oxide reacted with the IL and deposited on the surfaces in contact. An example of a chemical reaction of CuO with a water-free [NTf2] anion-based IL was represented in the work [37]. The authors pointed out that CuO could successfully be dissolved in the water-free IL upon heating to 175 °C for 24 h. Since the wear test conditions in the present work were the temperature of 60 °C and duration of 2 h, the CuO nano-oxide could chemically react with the [N1888] [NTf2] but not dissolve in the IL. As it can be seen from Figure 2 and Figure 4, the copper oxide combined with the IL presented a higher wear width value than the pure IL and the IL + ZnO nanoparticles, but a lower coefficient of friction than the pure [N1888] [NTf2]. The decrease in friction is due to the CuO nanoparticles compensating for the mass loss on the wear scar surfaces, which is verified by the presence of copper in the EDX analysis results. Hence, the lubrication mechanism of CuO nanoparticles when used as additives in the IL is tribo-sintered on the wear surfaces.

The wear mechanism of CuO nanoparticles combined with the IL depends on the concentration of CuO. The test lubricated with IL + 0.2 wt% CuO presented a lower friction coefficient and wear scar width than the test lubricated with IL + 0.5 wt% CuO. The weight percentage of Cu on the wear surfaces from the EDX analysis results for 0.5 wt% CuO test was lower than the 0.2 wt% CuO test. It is speculated that the lubrication mechanism of CuO nanoparticles may be more rolling effects than mending the surfaces, as the concentration of CuO increases.

For ZnO nano lubricants, almost no Zn component was found from the EDX analysis on the worm surfaces. Thus, the zinc oxide nanoparticles with spherical shape could act as third body mechanism with pure rolling effects. In the third body mechanism, the ZnO nanoparticles are considered nano ball bearings. They could roll among the metal surfaces, and transfer sliding friction into rolling friction. With rolling friction, the contact areas and shear stress at contact decrease, which leads to the reduction in friction coefficient and wear. This rolling effect of the third body mechanism in friction reduction has been proven by theoretical [38] and experimental results [20,25,39]. In summary, the lubrication mechanisms of CuO and ZnO nanoparticles in the ionic liquid are tribo-sintering and third body with rolling effects, respectively.

The EDX analysis on the chemical composition of the defects shows a high oxygen concentration compared to those of scratches. This observation confirms that oxidative wear occurred during the experiments. Oxidation is considered a major cause of defects. In Figure 9, the intensity ratio of O/C is surveyed by the O spectra at near 750 eV and the C spectra at near 350 eV. It can be seen that the ratio of O/C increases in the tests lubricated with ZnO nanoparticles. The increase in O/C ratio suggests that the breaking of the C-O bonds has occurred [40], and that the C-O bonds formation can increase oxidation state [41]. Thus, the severity of defects depends on the formation of C-O bonds on the metal surfaces. In the tests with zinc oxide nanoparticles, the number of defects on the wear surfaces was less (see Figure 5) but the severity of defects may be higher upon heating than those of the tests with copper oxide nanoparticles.

## 4. Conclusions

In this study, the effects of two oxide nanoparticles, CuO and ZnO, on the lubrication mechanism of the IL [N1888] [NTf2] in a steel-to-steel contact were investigated by performing the wear tests on the MTM tribotester. The morphology and chemical components on worm surfaces were analyzed by the optical microscope and SEM/EDX. From the obtained results, the lubrication mechanisms of the oxide nanoparticles in IL, including rolling, mending, and tribofilms’ formation of nanoparticles on the contact surfaces are discussed in detail to explain the reduction in friction coefficient and wear. The conclusions from the present study are summarized as follows:

(1) At the same concentrations of 0.2 and 0.5 wt%, the ZnO nanoparticles enhanced the tribological behavior of IL better than the CuO nanoparticles.
(2) Compared to the test lubricated with the IL, the addition of both oxide nanoparticles in the IL could reduce the friction coefficient by approximately 2.4% to 3.5% depending on their concentrations. However, the wear width was decreased by 4.6% to 32% in the experiments with ZnO, while it was increased by 8.6% to 23.7% in the experiments with CuO. Hence, ZnO nanoparticles in the IL show a better result in reducing wear width than the CuO nanoparticles. This behavior could be related to the fact that CuO nanoparticles have a lower bulk hardness than ZnO and are thus more likely to be deposited and flattened at the contact area, resulting in a larger interface for friction [34].
(3) From the EDX analysis results, it can be seen that the lubrication mechanisms of CuO and ZnO, when used as additives in the IL, are tribo-sintering and third body with pure rolling mechanisms, respectively.
(4) Out of the two types of nanoparticles and two concentrations tested, the best tribological performance was observed at the concentration of 0.2 wt% ZnO.

## Figures and Tables

**Table 1:** MTM specimen materials and tests conditions.

Material Properties	Specimens	
	Ball	Disc
Material	AISI 52100 steel	AISI 52100 steel
Hardness	800–920 HV	720–780 HV
Surface roughness	Ra 0.02 μm	Ra 0.02 μm

**Table 2:** AISI 52100 steel chemical composition properties [31].

Element	Fe	Cr	C	Mn	Si
Weight (%)	96.50–97.32	1.30–1.60	0.98–1.10	0.25–0.45	0.15–0.30

**Table 3:** Chemical composition and properties of the tested ionic liquid.

Name (CAS Number)	Methyltrioctylammonium Bis(Trifluoromethylsulfonyl)imide (375395-33-8)				
Properties	Cation [N_1888_]	Anion [NTf_2_]	Purity (%)	Viscosity 40 °C (cSt)	Molecular Weight
	C_25_H_54_N^+^ 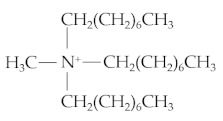	C_2_F_6_S_2_O_4_N^−^ 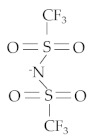	99	200.7	648.85

**Table 4:** Distribution of chemical components on scratches.

Element	IL	IL + 0.2 wt% CuO	IL + 0.5 wt% CuO	IL + 0.2 wt% ZnO	IL + 0.5 wt% ZnO
Fe	83.8	81.4	81.4	83.3	85.0
C	10.9	11.5	13.6	12.9	10.6
O	2.0	3.2	2.1	1.1	1.0
F	1.8	1.7	0.5	0.8	1.7
Cr	1.1	1.2	1.1	1.3	1.3
Si	0.4	0.5	0.6	0.4	0.5
S	0.1	0.0	0.0	0.0	0.0
Cu	0.0	0.4	0.7	0.0	0.0
Zn	0.0	0.0	0.0	0.0	0.0

## Data Availability

The study did not report any data.
